# Supplementary material for: Protracted development of gaze behaviour
Source: Nat Hum Behav. 2025 Jun 5;9(9):1887–97. doi: 10.1038/s41562-025-02191-9 (PMC12454113; doi:10.1038/s41562-025-02191-9)
Supplement: Supplementary file 1 — Supplementary Figs. 1–10, Methods and Results. [file 41562_2025_2191_MOESM1_ESM.pdf]

nature human behaviour

Supplementary information https://doi.org/10.1038/s41562-025-02191-9

Protracted development of gaze behaviour

In the format provided by the authors and unedited

Supplementary Materials

Supplementary Methods

Procedure

Figure S1 gives an overview of all phases of the experimental procedure. Participants took their seats in front of a touchscreen panel and selected their preferred language (either German or English). Following this, they provided informed consent and indicated their age, gender, whether they had participated before (yes or no), and whether they wore glasses (yes or no). To ensure accurate positioning, a customized position guide offered real-time feedback to confirm that the participant was within the trackable range. Subsequently, participants completed a 5-point calibration and validation procedure. If the average validation accuracy was less than 0.7 degrees of visual angle (dva), the procedure was repeated once more. After this, participants watched an instructional video welcoming them to the exhibit and instructing them to ‘look at the images in any way they want’. Following these instructions, 41 images were displayed sequentially, each presented at the centre of the screen for 3 seconds, with a central fixation cross displayed between trials. The image set consisted of the OSIE40[1], which was the basis for all analyses and an additional image of the museum which was used for gaze illustration later (see below). A trial would not commence before the participant's gaze was within 2 dva. from the fixation cross for 1 second. The presentation order of images remained the same for all participants. Upon completion of the free viewing task, participants saw a heatmap of their personal gaze on an image of the museum (image 41) and information about how their average fixation duration compared to that of others. Finally, they saw a display with a certificate of participation. After that they could chose to engage in another ’eyetracking game’ or end participation.

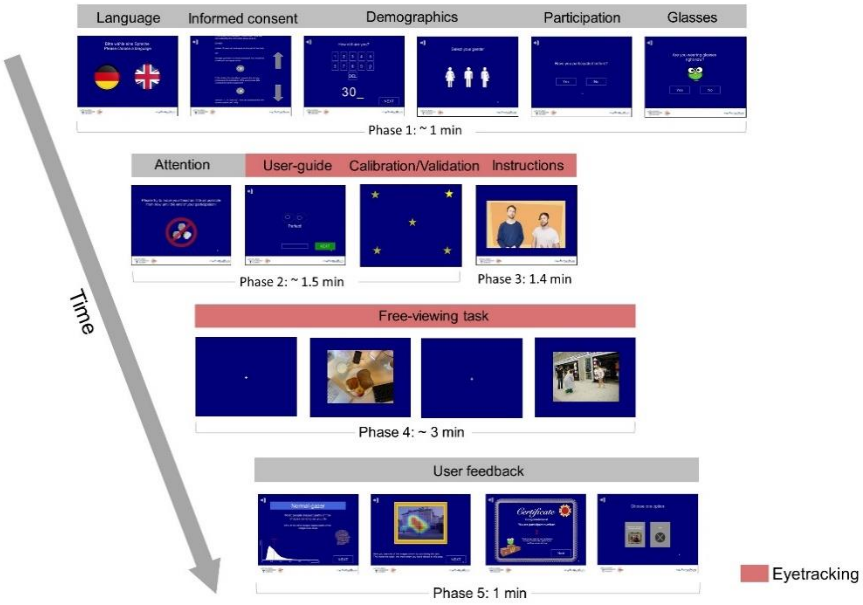

Figure S1. Phases of experimental procedure. Each frame shows a screenshot from a different phase of the experiment. Red bars indicate phases during which eye-tracking data was recorded. Feedback screens were based on online analyses of data and participant-specific.

Validation accuracy

To probe the accuracy of the present eye-tracking data, we computed the average validation accuracy in dva for each individual, as well as the median validation accuracy for each age group. This was repeated once for all collected datasets, and once for those included in the present analyses (i.e. those with a calibration error < 1 dva).

Protracted development of text salience independent of English proficiency

To test whether the protracted development of text salience in our mostly German-speaking sample hinges on (lacking) English language proficiency, we conducted two additional analyses. First, we selected the subsample of participants who chose English as their preferred language (n = 426) and determined their dwell time proportion towards text across age bins. Next, we computed the dwell time proportion towards text in the full sample, but limited it to two images that included words and numbers common in both languages. As in the main analysis, we computed the corresponding averages for each age bin, fitted

polynomials to the resulting developmental trajectory and adopted the sliding window approach to pinpoint periods of linear change (see main methods).

Gender differences in semantic salience across age

To test gender differences in gaze towards objects of several semantic attributes, we determined the proportion of dwell time and first fixations devoted to text, faces, touched objects, and bodies for male and female participants across different age groups. We then computed two-sample t-tests between male and female participants for each age group and semantic category. P-values were Holm-Bonferroni adjusted for all 34 tests within each semantic category. In the second step, we conducted two-sample t-tests between males and females for each semantic category, pooled across all age groups. Here, Holm-Bonferroni adjustments were applied to all tests considering the dwell time proportion and first fixation proportion, respectively (4 tests each).

Protracted development of horizontal bias in images without text

To test whether the protracted development of the horizontal bias is tied to text salience, we replicated our analyses of saccadic directions for the subset of 17 images that contained no text elements (see main methods).

Protracted development of visual exploration using a larger window size

To probe a more gradual decline of visual exploration with age, we again used a sliding window approach to test periods of broad linear change for the centre bias, number of objects fixated, fixation frequency and intra-individual entropy across age, however this time using a window size of 10 and extending the analysis to include the full age range, including bins with smaller sample sizes at higher ages.

Cross-validation of curve fitting across participants and images

One motivation for using curve fitting in the present study is the assumption that curve-fitted trajectories more closely reflect true developmental trends, which we expect to be smooth and replicable, while observed bin-by-bin deviations from smooth trends are likely

unreliable. To test this assumption for our present data, we first compared the replicability of fitted and observed trajectories across independent sets of participants. Specifically, we examined trajectories for dwell time and first fixation proportions for four semantic categories (text, faces, touched objects, and bodies), as well as for the centre bias, horizontal bias, visual exploration, and intra-individual entropy. We first identified the most parsimonious model for each developmental trajectory, once using data from all odd-numbered participants and then again using data from all even-numbered participants (see the methods section for a detailed explanation of how the best-fitting model was selected). Then we correlated these fitted trajectories across odd and even participants. Similarly, we calculated the observed bin-wise mean values for each metric separately for odd- and even-numbered participants and correlated those with each other.

Moreover, we tested whether the curve fits for odd-numbered participants generalise to the observed means from even-numbered participants. Following Spearman[2], we estimated the noise ceiling for correlations between fits and empirical data as the geometric mean of their respective split-half reliabilities (see above).

Finally, we fitted separate developmental trajectories for data from odd and even scenes and tested their reliability in the same way. Again, we calculated the correlations between the fitted values for odd and even scenes, as well as the correlations for the corresponding observed bin-wise means. We also tested whether the curve fits for odd scenes generalise to the observed means for even scenes.

Robustness of scene selection

To test the generalizability of the applied stimulus set, we harnessed previously published eye-tracking data from n = 101 adult observers who participated in a free viewing task in two separate sessions, about one week apart (see Linka et al., 2020 for more information). During the first session, participants viewed 700 images[3], with the initial 40 images being the ones used in our current study. On the second day of testing, the same

participants viewed 200 images, starting with the same 40 images displayed in a shuffled order. To examine the generalizability of gaze biases for our current stimulus set to those for other scenes, we calculated the correlation between individually estimated gaze parameters for our stimulus set versus those observed for the same participants viewing 660 other scenes. Note that this probes the robustness of individual estimates, which is a highly conservative test regarding the metric we used here: age specific averages.

Reliability of means and effects of stimulus order

To test the reliability of group means and investigate a potential image order effect, we empirically determined the re-test error of observed means for adults completing the free-viewing experiment (OSIE 40) twice. For one sample, the stimulus order was constant across testing days, for the other it was shuffled during the re-test.

For the shuffled condition, we re-analysed previously published data (Linka et al., 2020). In that study, n = 101 adults participated in a free-viewing experiment conducted over two sessions one week apart. During the first session, participants viewed 700 images, with the initial 40 images being the ones included in our current study. On the second testing day, the same participants viewed 200 images, starting with the same 40 images, now presented in a shuffled order. To compute the re-test error for a given metric, we computed the absolute difference for the respective group mean between testing days.

For the adult sample viewing stimuli in a constant order on both days, we conducted a new experiment and calculated re-test errors in the same way (see below for details on experimental methods). We then compared re-test errors for shuffled and constant stimulus orders to the observed size of developmental changes in our main sample. Observed developmental changes were defined as the difference between the peak and minimum values of each fitted developmental trajectory (bin-wise mean values; n = 6720).

Supplemental stimulus order experiment: Subjects

A total of 397 datasets were collected at Justus Liebig University from n=223 participants (M_{age} = 26.33, SD =8.66) of whom 137 identified as female (61%), 84 as male (38%), and 2 as diverse (1%). Of these, 18 datasets were excluded due to an average validation error exceeding 1 dva. Additionally, 10 datasets were removed, because they contained incomplete data for more than half of the 40 presented images. Finally, 47 datasets were discarded as participants attended only one session. This resulted in a final sample of 322 datasets from 161 participants, with testing sessions separated by an average of 5.9 days (range: 3 to 13 days). The study received approval from the Local Ethics Committee of the Department of Psychology (Fb06) at Justus Liebig University Giessen (Ethics Protocol number: LEK FB06 2018-0051). All participants were recruited on campus and provided written informed consent before the study began.

Supplemental stimulus order experiment: Apparatus & stimuli

To closely replicate the setup of the main study, we developed and made use of two mobile eye-tracking stations (see Figure S2 for an image of one setup) that used hardware and software tightly matched to those used in the eye-tracking exhibit for the main study. Eye movements were recorded binocularly using a Tobii Pro Spark Eye Tracker (Tobii AB, Danderyd, Sweden) operating in head-free, remote mode at a distance of 50-90 cm with a sampling rate of 60 Hz. The median validation accuracy for this study was 0.39 d.v.a. (SD =1).

Supplemental stimulus order experiment: Procedure

The procedure closely followed that of the main experiment. The main difference was that participants entered a personalised ID, which enabled matching of datasets across testing days. Also, participants received instructions in person rather than via a pre-recorded video. Participants sat at an average distance of 63 cm from the screen. After completing the first session, participants received a document detailing the earliest date (minimum of 3 days later) they should return to for their second testing session. The presentation order of images was consistent across all participants and between testing sessions. After completing the second testing session, participants were compensated with 10 euros.

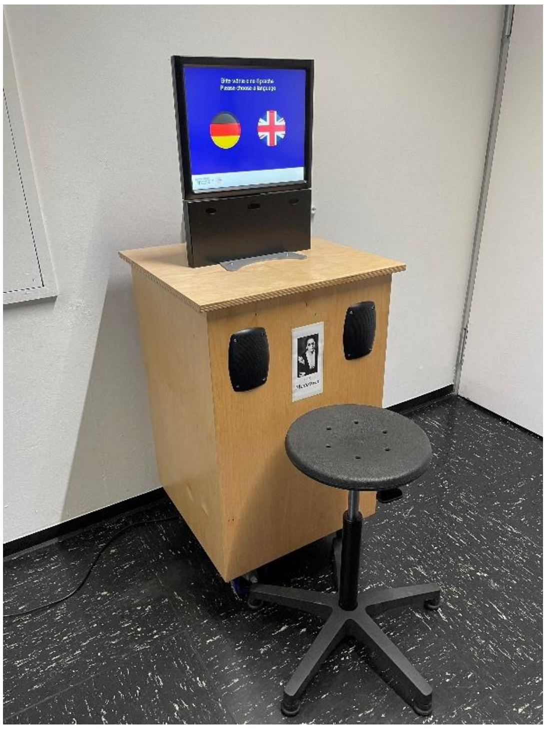

Figure S2. Eyetracking station set up. Image of one of the two eyetracking stations used for collecting eyetracking data. To facilitate consistent reassignment of participants to the same station across testing sessions, the stations were labeled with distinct names: ‘Lise Meyetner’ (visible on the station's label) and ‘Albert Eyenstein’.

Data Processing

All pre-processing and statistical analyses were identical to the main experiment.

Supplementary Results

Validation accuracy

Figure S3 panels a and b show the median validation accuracy per age group for the included (green) and excluded (red) data in our study. The entire collected sample of 13,984 datasets has a median validation accuracy of 0.6 dva. For all analyses in this study, we used an individual threshold of <1 dva as the inclusion criterion for further processing. Using this criterion, the remaining datasets show a median accuracy of 0.47. When computing the validation accuracy per age group (bin), we observe weaker validation accuracies around the age groups 5-6 to 13-14 (median = 0.78 dva) for all collected datasets. Importantly, when only analysing included participants with a mean validation accuracy <1 dva, this difference disappears (median = 0.49 dva).

Protracted development of text salience independent of English proficiency

Figure S4 shows the developmental trajectory of dwell time on text (1) when only including images containing text elements common in the German and English language and (2) when testing the English-preferring subsample. Our analyses replicated evidence for a protracted increase of text salience until age 15-16 (ΔAIC > 5). The development of text salience for the two images with numbers and words common in German was best fitted by a 5th degree polynomial. The development of text salience in participants indicating English as their preferred language by a 2nd degree polynomial, with a clear overall protracted trend, which however was only significant between ages 7-8 and 9-10 as well as between ages 17-18 and 19-20 (ΔAIC >5), most likely to the drastically reduced sample sizes.

Gender differences in semantic salience across age

Next, we examined gender differences in semantic salience. First, we calculated differences between males and females for each age group and semantic category included in our study. Figure S5 shows the age trajectories for semantic salience, indicated by dwell time proportion (a) and first fixation proportion (b) for males and females, respectively. Panel c displays the overall gender difference (pooled across age groups) for all included semantic metrics. Overall, our findings show similar developmental trajectories for male and female

participants. For text, males showed a somewhat smaller proportion of dwell time in the age group 9-10, $t(746) = -3.43$, $p < .05$, $d = -0.25$. For faces, male participants spent a somewhat larger proportion of dwell time and first fixations in the age groups 11-12 (first fixation:

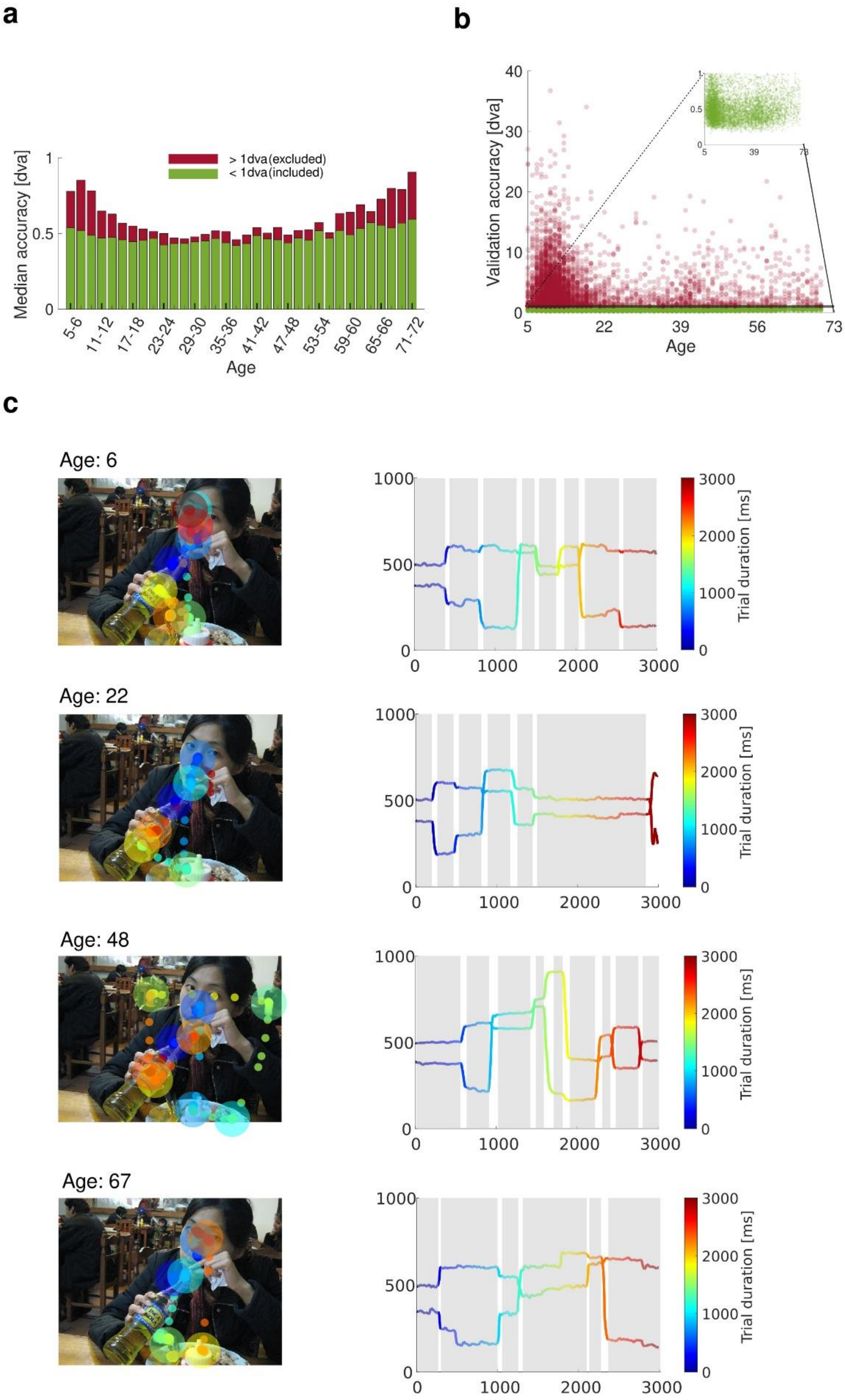

Figure S3. Eye-tracking validation accuracy across age and example trace plots. Panel a shows a bar plot displaying the median accuracy in degrees of visual angle (dva) for each age bin. Values in green represent values computed including participants with an accuracy of less than 1 dva, whose data were included in all analyses of this study. Values in red represent median scores that include data from participants with a mean accuracy score greater than 1 dva. Please note that each age bin spans two years. Due to space constraints, only every third tick is labelled, and these are marked in bold for easier identification. Panel b displays the individual mean validation accuracy scores in dva across age. Each scatter point represents one individual. Green scatter points indicate participants included in all analyses, with a validation accuracy of less than 1 dva, highlighted in the zoomed-in scatter plot on the upper right. Red scatter points represent participants with a mean validation score greater than 1 dva. Panel c displays example trace plots for free viewing data**.** The overlay plots on the left show the raw eye-tracking data as scatter points (90Hz). Transparent disks indicate fixation events. Each row shows the data of an example participant from a different age group. The plots on the right show the corresponding horizontal and vertical position traces as interpolated lines connecting data points, with grey overlays marking periods classified as fixations. Each data point is colour-coded to represent the trial time, ranging from 0-3000 ms, as indicated by the colour bar on the right. Fixation and saccade classifications clearly map onto the data traces, but two fixations separated by a very small saccade may be classified as a single fixation.

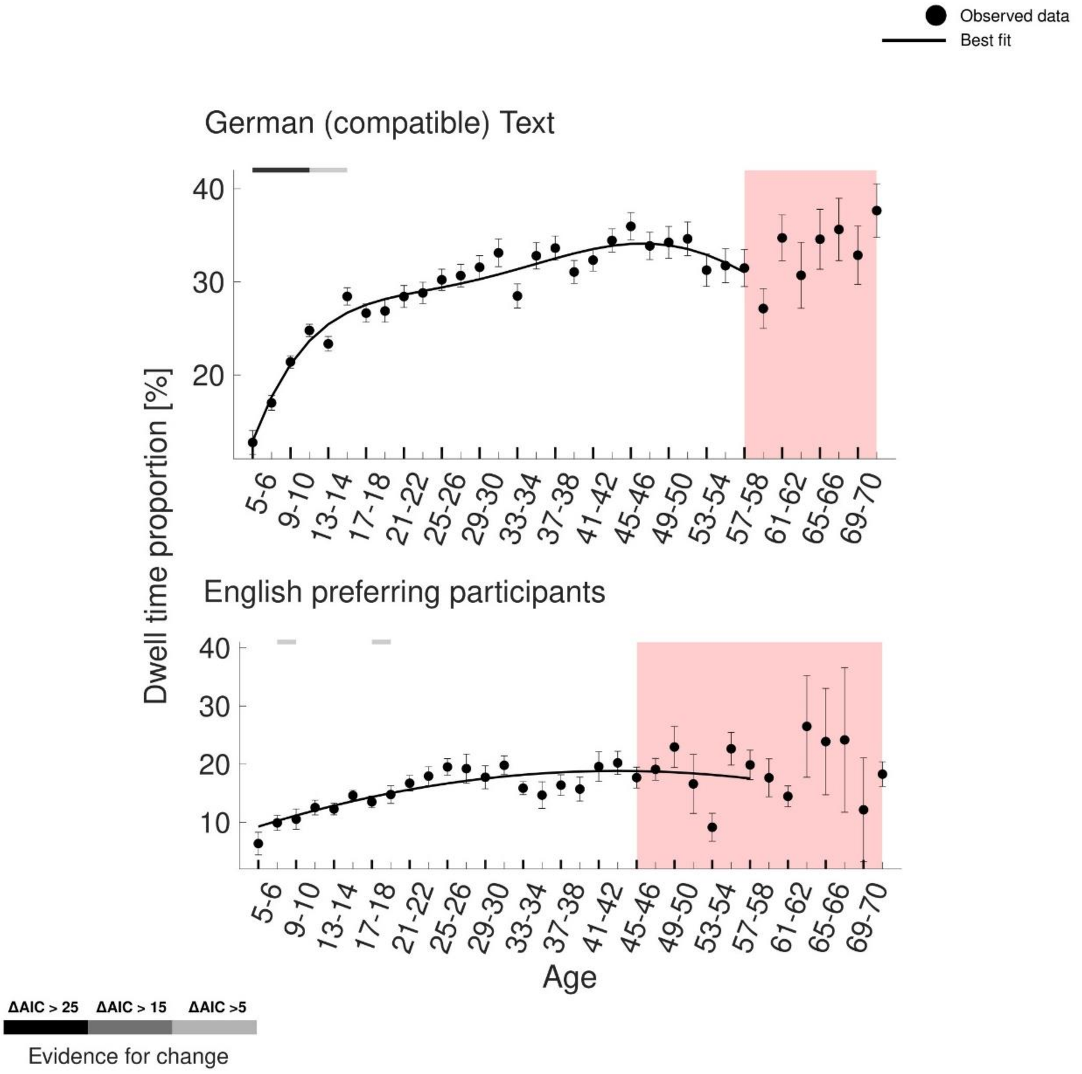

Figure S4. Protracted development of semantic salience. Scatter points display the mean proportion of dwell time towards text elements (y-axis) across age bins from ages 5-6 to 71-72 (x-axis) for images containing text elements common in German and English (upper panel) analysing data from a total of $n = 6720$ participants and for the English-preferring sample ($n = 426$; lower panel). Please note that each age bin spans two years. Due to space constraints, only every second tick is labelled, and these are marked in bold for easier identification. Error bars indicate +/- 1 standard error of the mean (SEM), black lines best polynomial fits. The horizontal lines at the top of a given figure indicate the level of evidence for a given bin to be part of broad linear change via shades of grey, as shown in the inset (sliding window analysis, cf. main text and methods). Red patches mark age bins with lower sensitivity to detect developmental trends ($n < 90$ per bin in upper panel; $n <10$ per bin in lower Panel).

$t(793) = 4.29$, $p < .001$, $d = 0.31$; dwell time: $t(793) = 5.29$, $p < .001$, $d = 0.38$) and 15-16 (first fixation: $t(309) = 3.34$, $p < .05$, $d = 0.39$; dwell time $t(309) = 3.48$, $p < .05$, $d = 0.40$). Further males showed a larger proportion of dwell time in the age group 13-14 (dwell time: $t(529) = 5.58$, $p < .001$, $d = 0.49$). Regarding touched objects, males spent a smaller proportion of dwell time than females in the age groups 5-6 ($t(138) = -3.43$, $p < .05$, $d = -0.58$), 11-12 ($t(793) = -3.85$, $p < .01$, $d = -0.28$), and 15-16 ($t(309) = -3.29$, $p < .05$, $d = -0.38$), and first fixations in the age group 69-70, $t(31) = -4.31$, $p < .01$, $d = -1.5$. For body salience no age-specific differences were found. All other age-wise comparisons were not significant (all $p > 0.05$). Finally, we examined gender differences in dwell time and first fixation proportions, pooled across all age groups. For text, we found that males spend a smaller proportion of dwell time compared to females ($t(6520) = -2.5$, $p < .05$, $d = -0.06$), but did not differ in terms of first fixations ($t(6520) = 0.99$, $p = .32$, $d = 0.02$). Males showed slightly higher face salience than females (First fixations: $t(6520) = 6.19$, $p < .001$, $d = 0.16$; Dwell time = $t(6520) = 9.01$, $p < .001$, $d = 0.23$) and slightly lower salience for touched objects (first fixation: $t(6520) = -3.04$, $p < .01$, $d = -0.08$; dwell time $t(6520) = -4.72$, $p <.001$, $d = -0.12$). Lastly, we found that overall, males spent a smaller proportion of their dwell time ($t(6520) = -2.62$, $p < .05$, $d = -0.07$) and first fixations ($t(6520) = -4.02$, $p < .001$, $d = -0.10$) on bodies .

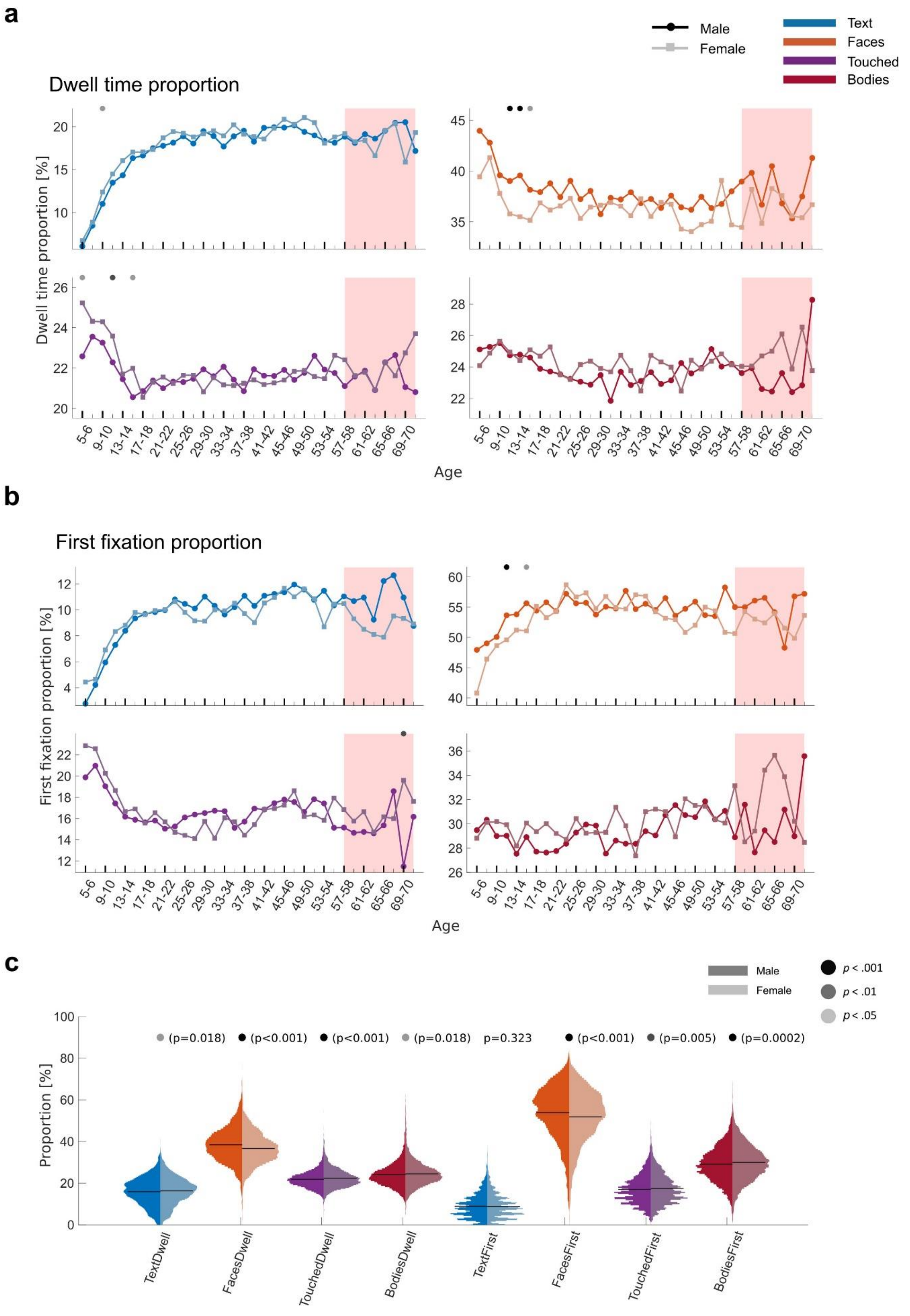
a
Male
Female
Text
Faces
Touched
Bodies
Dwell time proportion
Dwell time proportion [%]
Age
b
First fixation proportion
First fixation proportion [%]
Age
c
Male
Female
p < .001
p < .01
p < .05
Proportion [%]
(p=0.018) (p<0.001) (p<0.001) (p=0.018) p=0.323 (p<0.001) (p=0.005) (p=0.0002)
TextDwell
FacesDwell
TouchedDwell
BodiesDwell
TextFirst
FacesFirst
TouchedFirst
BodiesFirst

Figure S5. Gender differences in semantic salience. Line plots with scatter points display the mean proportion of dwell time (a) and first fixations (b) towards text, faces, touched objects and bodies (y-axis) across age bins from ages 5-6 to 71-72 (x-axis) for females (square markers and desaturated colours) and males (point markers). A total of 6,521 participants were included in the analysis. Of this sample n = 2,737 identified as female and n = 3,784 as male. The two-tailed P values were computed based on two-sample t-tests between male and female participants for each age group and semantic category. P-values were Holm-Bonferroni adjusted for all 34 tests within each semantic category. Please note that each age bin spans two years. Due to space constraints, only every second tick is labelled, and these are marked in bold for easier identification. Semantic categories are indicated by colours, as shown in the inset. Red patches mark age bins with lower sensitivity to developmental trends (n < 40 per bin for females; n < 50 per bin for males). Panel c shows violin plots depicting the distributions of proportion of dwell time and first fixations for males (left hand leaves) and females (right hand leaves, desaturated colours) pooled across all age groups. Here, two-tailed p-values were calculated using two-sample t-tests comparing male and female participants for each object category and metric. The p-values were Holm–Bonferroni adjusted across the four tests for dwell time proportion and first fixation proportion, respectively. Black lines overlaid on each leaf indicate the mean. The error shades indicate +/- 1 standard error of the mean (SEM). Please note across all panels, the points above the lines indicate the level of significance for a given gender difference via shades of grey.

Protracted development of horizontal bias for images without text

Polar histograms replicated a strong increase in horizontal bias from ages 5-6 to 13-14 for the subset of images excluding text (Figure S6a). The same was true for the developmental trajectory of the proportion of horizontal saccades, which steeply increased from 47% to 52% between ages 5-6 and 13-14 (ΔAIC > 25), before increasing more moderately to 54% at age 15-16 (ΔAIC > 5) and finally stabilizing (Figure S6b). The corresponding developmental trajectory was best fitted by a 9th degree polynomial.

Protracted development of visual exploration using a larger window size

Next, we tested linear developmental changes in the centre-bias, indicated by the mean distance to the image centroid (Figure S7a), visual exploration as indicated by the absolute number of objects fixated (Figure S7b), fixation frequency (Figure S7c), and intra-individual fixation entropy (Figure S7d) via a sliding window approach using a window size of 10 bins and including higher age bins with small sample sizes and lower sensitivity (area of red patchesin Figure S7).

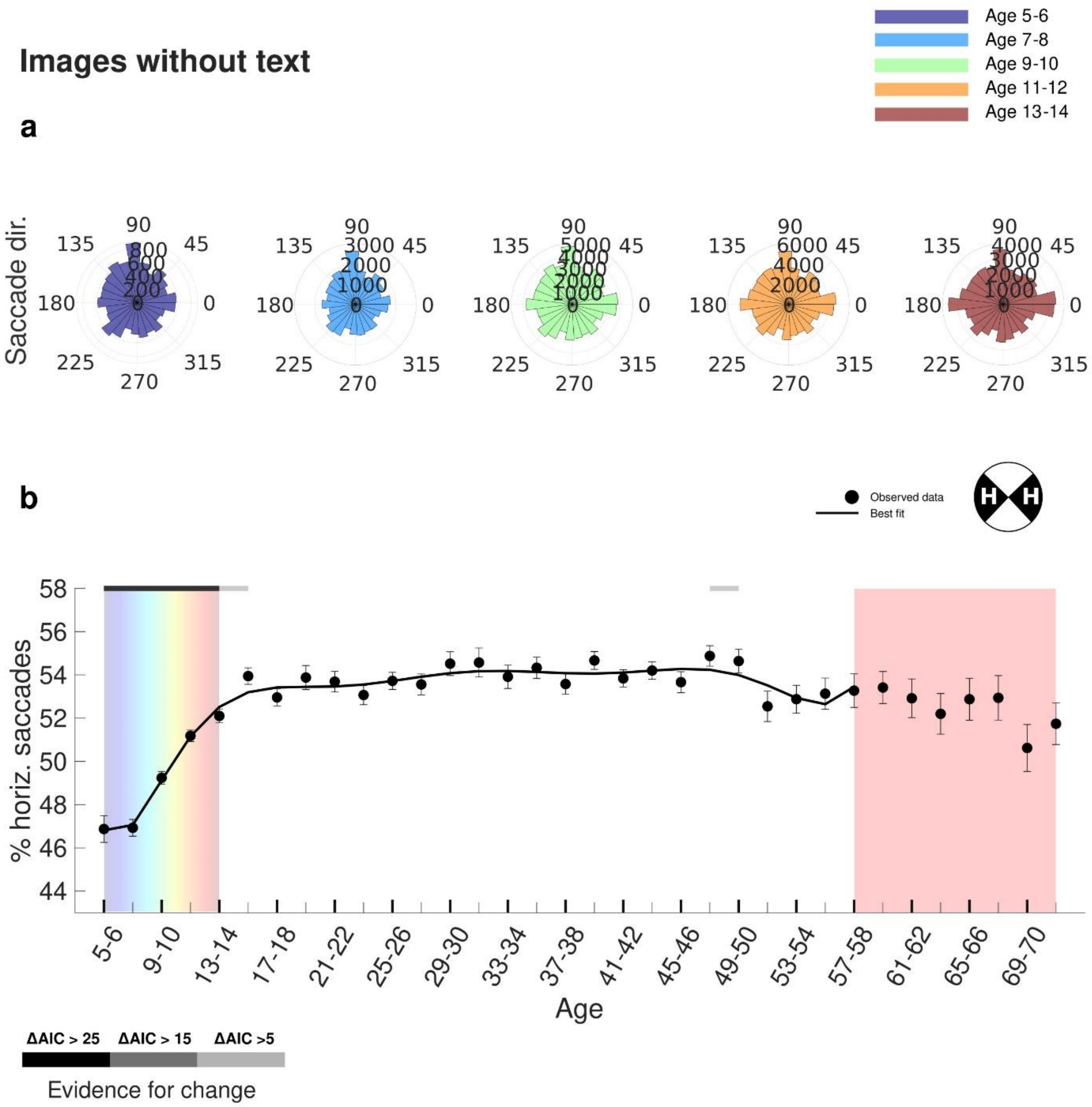

Figure S6. Protracted development of the horizontal bias for images excluding text. Panel a shows polar histograms for saccades across 36 direction bins from ages 5-6 to 13-14, as indicated by the colours shown in the inset. Panel b plots the developmental trajectory of the average proportion of horizontal fixations across age bins (scatter points). The error bars indicate +/- 1 standard error of the mean (SEM) and the black line the best fitting polynomial. The horizontal lines at the top of b indicate the level of evidence for a given bin to be part of broad linear change via shades of grey, as shown in the inset (sliding window analysis, cf. main text and methods). N = 6,720 participants were included in the analysis; for a more detailed distribution of participants across age groups, please refer to Figure 5 in the main text. Note that each age bin spans two years. Due to space constraints, only every second tick is labelled, and these are marked in bold for easier identification. The red patch marks age bins with lower sensitivity to developmental trends (n < 90 per bin).

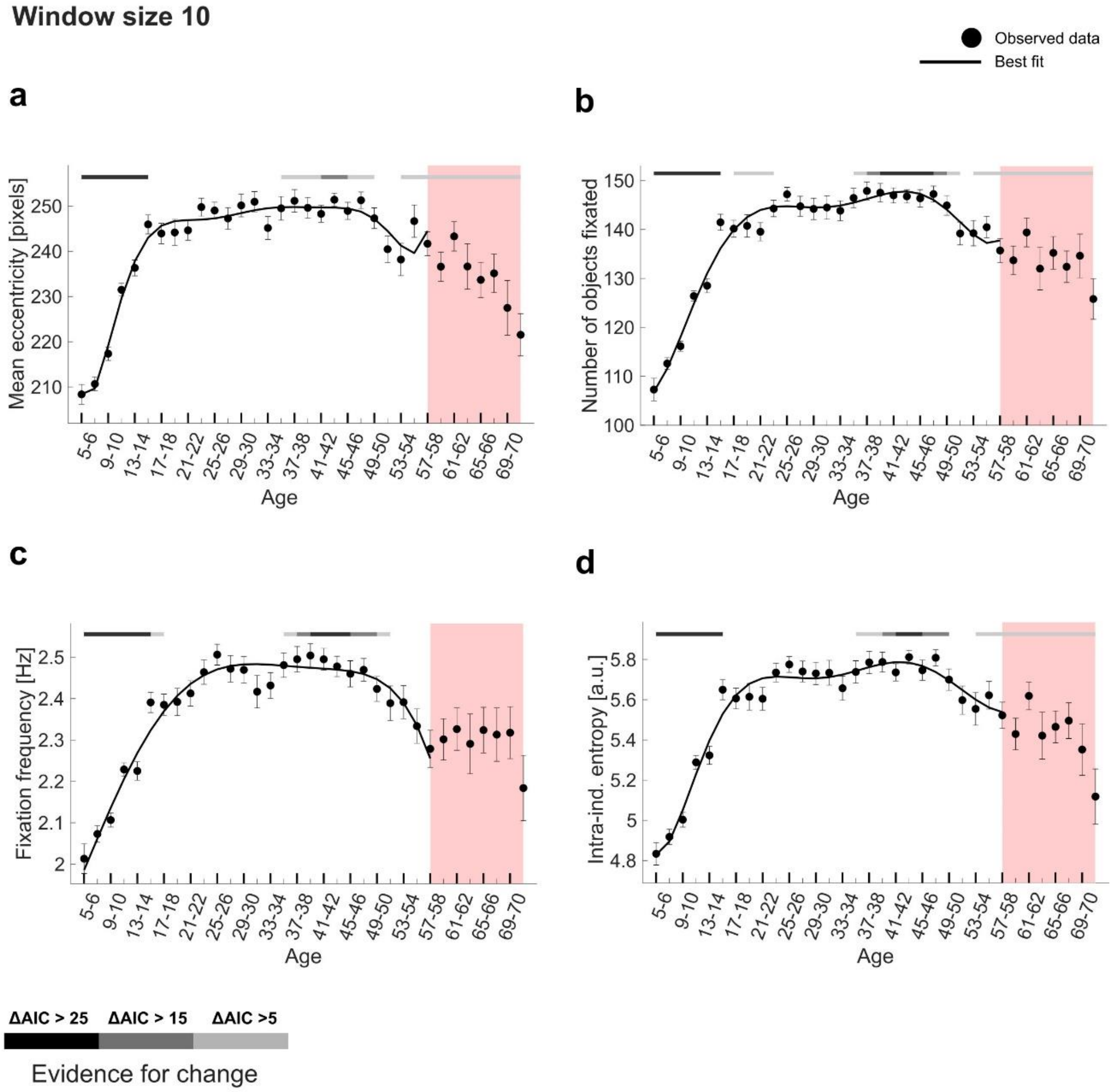

Figure S7. Protracted development of visual exploration. Scatter points illustrate the mean distance to the image centroid (in pixels; a), the average number of objects fixated (b), the average fixation frequency (Hz; c) and the average entropy of observer-specific fixation maps (d) across age bins ranging from ages 5-6 to 71-72 (x-axis). Error bars indicate +/- 1 standard error of the mean (SEM), black lines best polynomial fits. The horizontal lines at the top of a given figure indicate the level of evidence for a given bin to be part of broad linear change via shades of grey, as shown in the inset (sliding window analysis, cf. main text and methods). Please note that each age bin spans two years. Due to space constraints, only every second tick is labelled, and these are marked in bold for easier identification. N = 6,720 participants were included in the analysis; for a more detailed distribution of participants across age groups, please refer to Figure 5 in the main text. Red patches mark age bins with lower sensitivity to developmental trends (n < 90 per bin).

The average distance of fixations from the image centre increased from 208 to 246 pixels between ages 5-6 and 15-16 (ΔAIC > 25) and decreased from 250 to 222 pixels from ages 35-36, with significant evidence for this decline between ages 35-36 and 49-50 and

between ages 53-53 and 71-72 ($\Delta AIC > 5$). The number of fixated objects across all images increased steeply from 107 to 142 between ages 5-6 and 15-16 ($\Delta AIC > 25$) before increasing more moderately from 140 to 144 between ages 17-18 and 23-24 ($\Delta AIC > 5$). The trajectory then decreased from 147 to 126 between ages 35-36 and 71-72 ($\Delta AIC > 5$, except ages 51-54). Similarly, fixation frequency increased from 2.02 Hz to 2.38 Hz between ages 5-6 and 17-18 ($\Delta AIC > 5$) and declined from 2.48 Hz to 2.4 between ages 35-36 and 51-52 ($\Delta AIC > 5$). Finally, intraindividual entropy increased by 14% between ages 5-6 to and 15-16 ($\Delta AIC > 25$) and declined by 11% between ages 35-36 and 71-72 ($\Delta AIC > 5$, except ages 49-54).

Cross-validation of curve fitting across participants and images

Figure S8a presents the reliabilities of curve fits (dashed lines) and observed means (bars) across participants. The reliability of curve fits across participants was close to ceiling for dwell time proportions (text: $r(25) = 1$, $p < .001$; faces: $r(25) = 0.87$, $p < .001$; touched: $r(25) = 0.94$, $p < .001$; bodies: $r(25) = 0.99$, $p < .001$), first fixation proportions (text: $r(25) = 0.99$, $p < .001$; faces: $r(25) = 0.96$, $p < .001$; touched: $r(25) = 0.94$, $p < .001$; bodies: $r(25) = 0.97$, $p < .001$), as well as the centre bias ($r(25) = 0.97$, $p < .001$), visual exploration ($r(25) = 0.97$, $p < .001$), intra-individual entropy ($r(25) = 0.96$, $p < .001$) and the horizontal bias ($r(25) = 0.96$, $p < .001$). The reliabilities of observed means were still high, but somewhat lower for all tested metrics (dwell time proportions; text: $r(25) = 0.98$, $p < .001$; faces: $r(25) = 0.76$, $p < .001$; touched: $r(25) = 0.76$, $p < .001$; bodies: $r(25) = 0.43$, $p < .001$), first fixation proportions; text: $r(25) = 0.94$, $p < .001$; faces: $r(25) = 0.86$, $p < .001$; touched: $r(25) = 0.86$, $p < .001$; bodies: $r(25) = 0.57$, $p < .001$), as well as the centre bias ($r(25) = 0.94$, $p < .001$), visual exploration ($r(25) = 0.92$, $p < .001$), intra-individual entropy ($r(25) = 0.91$, $p < .001$) and the horizontal bias ($r(25) = 0.87$, $p < .001$).

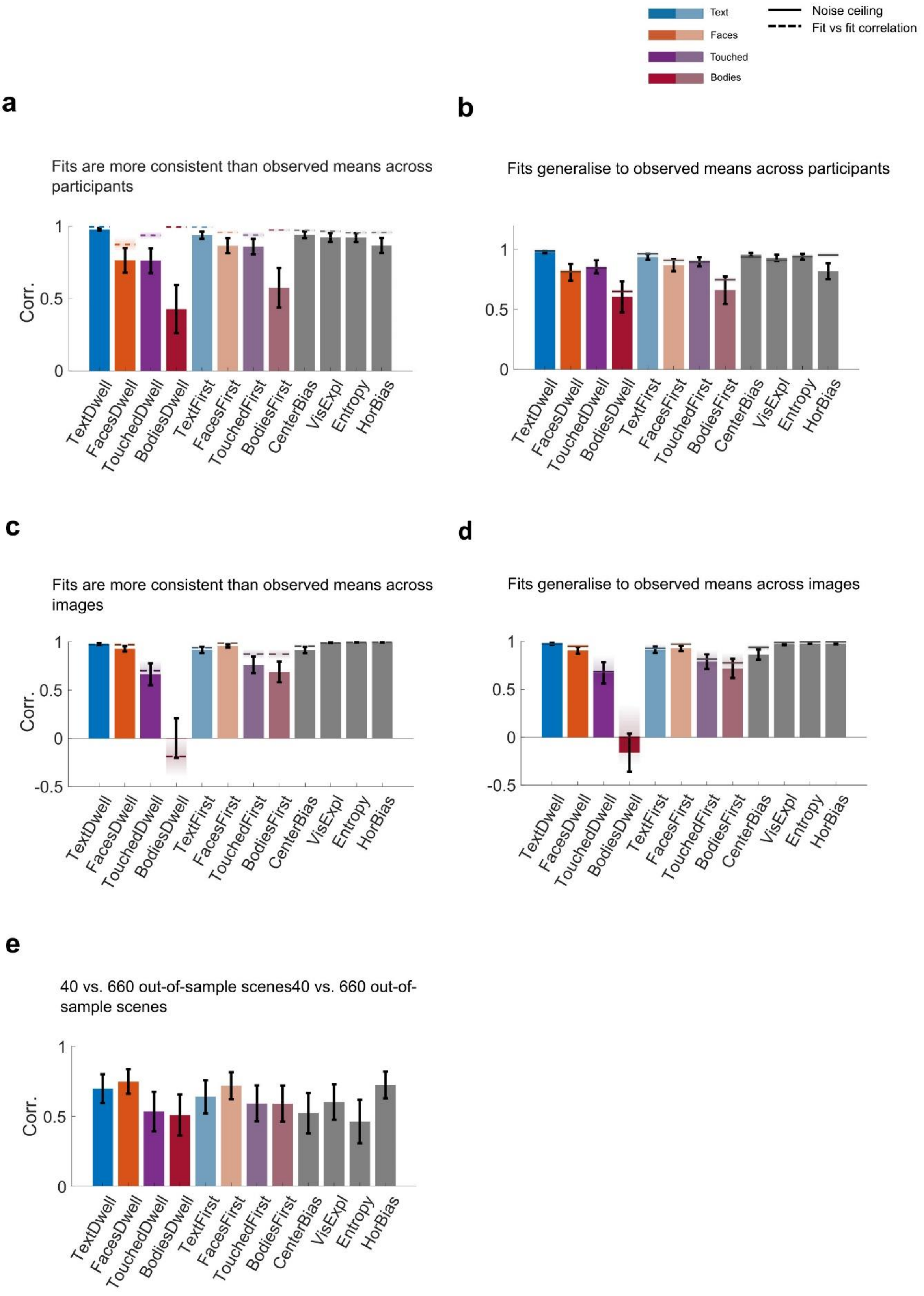
Text
Faces
Touched
Bodies
Noise ceiling
Fit vs fit correlation
a
Fits are more consistent than observed means across participants
b
Fits generalise to observed means across participants
c
Fits are more consistent than observed means across images
d
Fits generalise to observed means across images
e
40 vs. 660 out-of-sample scenes40 vs. 660 out-of-sample scenes
Corr.
TextDwell
FacesDwell
TouchedDwell
BodiesDwell
TextFirst
FacesFirst
TouchedFirst
BodiesFirst
CenterBias
VisExpl
Entropy
HorBias

Figure S8. Consistency of curve fits across participants and images and generalizability of individual differences to out of sample images . Panels a and b show the consistency of curve fits across participants**.** (a) Dashed lines show Pearson correlations between odd- and even-numbered participants for polynomial curve fits of developmental trajectories. Bar plots show the corresponding correlations without curve fitting (i.e. for observed bin-wise means), which are lower for all metrics. (b) Bar plots show cross-validated Pearson correlations between observed developmental trajectories (bin-wise means of even participants) and polynomial curve fits (fitted to bin-wise means from odd participants). All cross-validated correlations are close to the respective noise ceiling, which is defined as the average of the split-half consistencies shown in (a) and indicated by the horizontal lines. All error bars and error shades in a and b indicate standard errors of correlation estimates. Panels c and d show the consistency of curve fits across images. (c) Dashed lines show Pearson correlations between odd- and even scenes for polynomial curve fits of developmental trajectories. Bar plots show the corresponding correlations between observed trajectories (i.e. for observed bin-wise means). (d) Bar plots show Pearson correlations between observed developmental trajectories (bin-wise means of even scenes) and polynomial curve fits (fitted to bin-wise means from odd participants). All correlations are close to the respective noise ceiling, which is defined as the average of the split-half consistencies shown in (c) and indicated by the horizontal lines. All error bars and error shades in c and d indicate standard errors of correlation estimates. Panel e shows the generalizability of individual differences to out of sample images. Bars and error bars show Pearson correlation coefficients for individual differences in a given gaze metric, estimated based on the 40 images used in our study versus 660 other scenes. Error bars show 95% confidence intervals. Metrics are shown as labels below the x-asis and include dwell-time proportions on text, faces, touched objects, and bodies; the centre bias (mean eccentricity from image centroid), visual exploration (the number of distinct objects fixated), mean intra-individual entropy, and horizontal bias (measured by the proportion of horizontal saccades). Note that all correlations here pertain to the robustness of individual estimates, and that of central tendencies will be substantially higher (see main text).

Figure S8b shows the generalisation of fitted trajectories for data from odd participants to observed means from even participants, as well as the corresponding estimated noise ceilings. We observed large cross-validated correlations between fitted developmental trajectories and hold-out data for dwell time proportions (text: $r(25) = 0.98$, $p < .001$; faces: $r(25) = 0.81$, $p < .001$; touched: $r(25) = 0.86$, $p < .001$; bodies: $r(25) = 0.61$, $p < .001$), first fixation proportions (text: $r(25) = 0.94$, $p < .001$; faces: $r(25) = 0.87$, $p < .001$; touched: $r(25) = 0.90$, $p < .001$; bodies: $r(25) = 0.66$, $p < .001$), as well as the centre bias ($r(25) = 0.96$, $p < .001$), visual exploration ($r(25) = 0.93$, $p < .001$), intra-individual entropy ($r(25) = 0.94$, $p < .001$) and the horizontal bias ($r(25) = 0.82$, $p < .001$). All correlations were close to or exceeded their estimated noise ceilings (see Figure S8b). Together, this indicates the robustness of the applied curve fitting across particpants.

Similarly, Figure S8c presents the reliabilities of curve fits (dashed lines) and observed means (bars) across scenes. The reliability of curve fits across scenes was close to ceiling for most metrics showing developmental change, including dwell time proportions (text: $r(25) = .97$, $p < .001$; faces: $r(25) = 0.97$, $p < .001$; touched: $r(25) = 0.70$, $p < .001$; bodies: $r(25) = -0.19$, $p = .35$), first fixation proportions (text: $r(25) = 0.94$, $p < .001$; faces: $r(25) = 0.99$, $p < .001$; touched: $r(25) = 0.87$, $p < .001$; bodies: $r(25) = 0.87$, $p < .001$), as well as the centre bias ($r(25) = 0.96$, $p < .001$), visual exploration ($r(25) = 0.99$, $p < .001$), intra-individual entropy ($r(25) = 1$, $p < .001$) and the horizontal bias ($r(25) = 1$, $p < .001$). The reliabilities of observed means were the same or somewhat lower for all tested metrics (dwell time proportions; text: $r(25) = 0.98$, $p < .001$; faces: $r(25) = 0.93$, $p < .001$; touched: $r(25) = 0.66$, $p < .001$; bodies: $r(25) = 0.003$, $p = .99$), first fixation proportions; text: $r(25) = 0.92$, $p < .001$; faces: $r(25) = 0.96$, $p < .001$; touched: $r(25) = 0.76$, $p < .001$; bodies: $r(25) = 0.69$, $p < .001$), as well as the centre bias ($r(25) = 0.92$, $p < .001$), visual exploration ($r(25) = 0.99$, $p < .001$), intra-individual entropy ($r(25) = 1$, $p < .001$) and the horizontal bias ($r(25) = 1$, $p < .001$).

Figure S8d shows the generalisation of fitted trajectories for data for odd scenes to observed means for even scenes, as well as the corresponding estimated noise ceilings. We observed large cross-validated correlations between fitted developmental trajectories and hold-out data for dwell time proportions (text: $r(25) = 0.98$, $p < .001$; faces: $r(25) = 0.91$, $p < .001$; touched: $r(25) = 0.67$, $p < .001$; bodies: $r(25) = -0.16$, $p = 0.42$), first fixation proportions (text: $r(25) = 0.92$, $p < .001$; faces: $r(25) = 0.93$, $p < .001$; touched: $r(25) = 0.79$, $p < .001$; bodies: $r(25) = 0.72$, $p < .001$), as well as the centre bias ($r(25) = 0.86$, $p < .001$), visual exploration ($r(25) = 0.97$, $p < .001$), intra-individual entropy ($r(25) = 0.98$, $p < .001$) and the horizontal bias ($r(25) = 0.98$, $p < .001$). All correlations were close to or exceeded their estimated noise ceilings (see Figure S8b). Together, this indicates the robustness of the developmental trajectories across images.

Robustness to scene selection

Figure S8e shows that individual estimates based on the current stimulus set are generalizable to out-of-sample scenes (Dwell time proportions for text: r(99) = .70, p < .001; faces: r(99) = .75, p < .001; touched objects: r(99) = .53, p < .001; bodies: r(99) = .51, p < .001. First fixation proportions for text: r(99) = .64, p < .001; faces: r(99) = .72, p < .001; touched objects: r(99) = .60, p < .001; bodies: r(99) = .59, p < .001. Centre bias: r(99) = .52, p < .001; visual exploration: r(99) = .60, p < .001; entropy: r(99) = .46, p < .001; horizontal bias: r(99) = .72, p < .001). Please note, these estimates pertain to the generalisability of individual estimates to out-of-sample scenes, implying a substantially higher robustness of bin-wise group means (which are of interest in our current study).

Reliability of means and effects of stimulus order

To test the reliability of group means and a potential effect of image order, we compared the observed developmental change for each key metric in our main sample to the re-test error from adults completing the free viewing task twice, with either fixed or shuffled stimulus order during the second session. Figure S9 below shows developmental changes and retest errors for each metric and condition.

Our results show that retest errors for all tested metrics, which showed a developmental effect are orders of magnitude smaller than corresponding developmental changes. The following values indicate developmental changes (and re-test errors for fixed and shuffled image orders in brackets, separated with a comma): Dwell time proportions for text: 14.10 (1.5, 0.53); faces: 6.8 (0.62, 0.28); touched objects: 3.2 (0.07, 0.86); bodies: 1.97 (0.49, 0.31); First fixation proportions for text: 8.52 (0.63, 0.01); faces: 10.64 (1.7, 2.33); touched objects: 6.41 (1.94, 1.84); bodies: 2.46 (0.93, 2.45); Centre bias: 41.38 (0.52, 1.49);

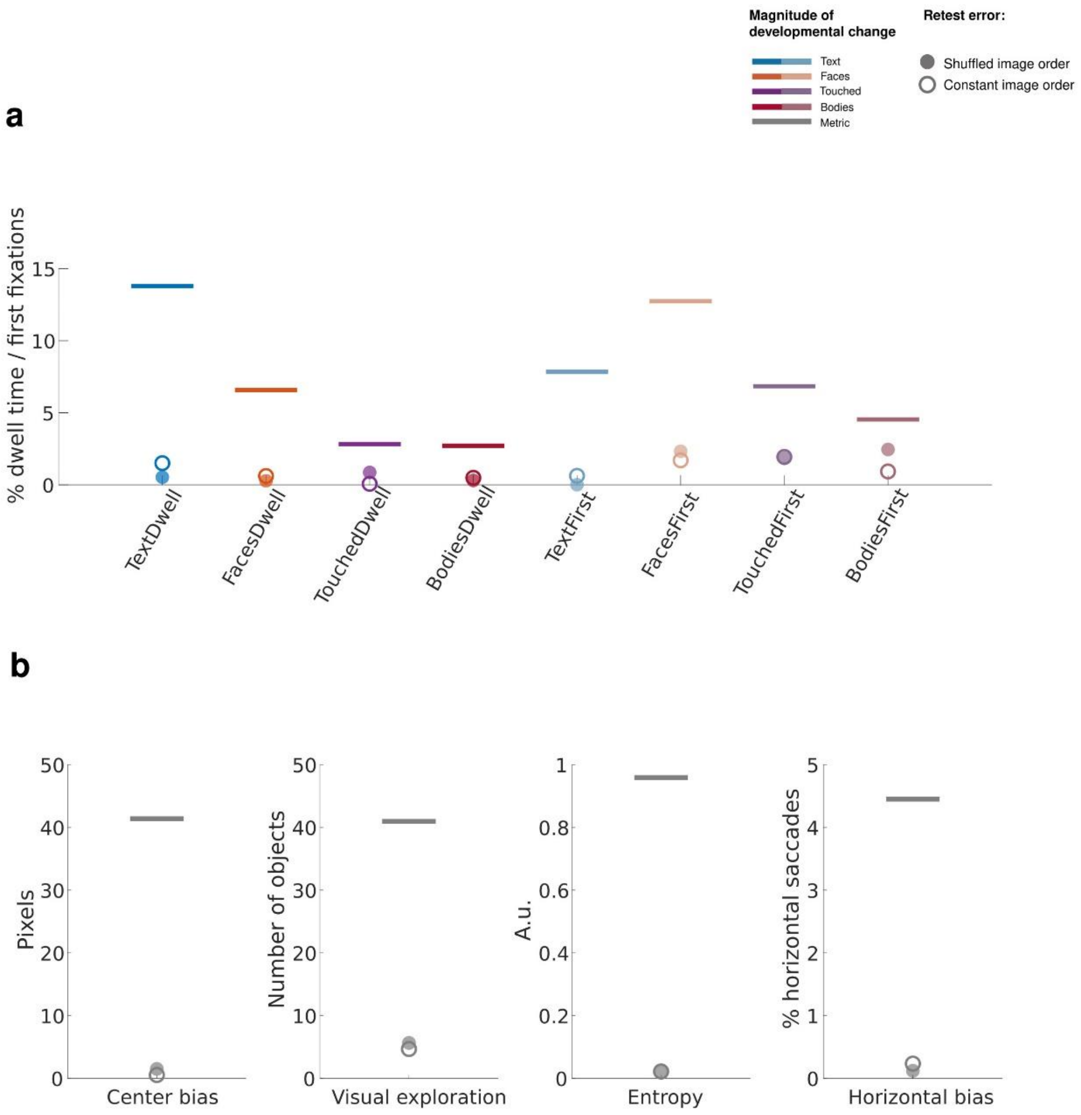

Figure S9. Developmental change and retest error for key metrics. Lines represent developmental change, calculated as the difference between the peak and minimum values of a given fitted developmental trajectory (based on data from n = 6720 participants; for a more detailed distribution of participants across age groups, please refer to Figure 5 in the main text). Filled dots and circles represent retest errors for shuffled and constant image orders, respectively. Retest errors were calculated as the absolute between-session difference of respective means for adult samples (322 datasets by n = 161 participants; see supplementary Methods for details). (a) shows developmental changes and re-test errors for average dwell time- and first fixation proportions across semantic dimensions, (b) for other key metrics as indicated below the x-axes.

visual exploration: 40.94 (4.66, 5.63); entropy: 0.96 (0.02, 0.02); horizontal bias: 4.45 (0.23, 0.12). Importantly, this was true independent of image order and there was no consistent effect of constant or shuffled image orders on re-test errors.

Robustness of gaze trajectories to participants wearing glasses

To probe whether the reported age trajectories are affected by participants wearing glasses, we recalculated developmental trajectories for all key metrics omitting participants who reported wearing glasses, resulting in a sample of n = 3996. Figure S10 shows age trajectories for dwell time (a) and first fixation proportions (b) across all included semantic object dimensions, and for visuospatial gaze biases (c). All results of the main analysis closely replicated.

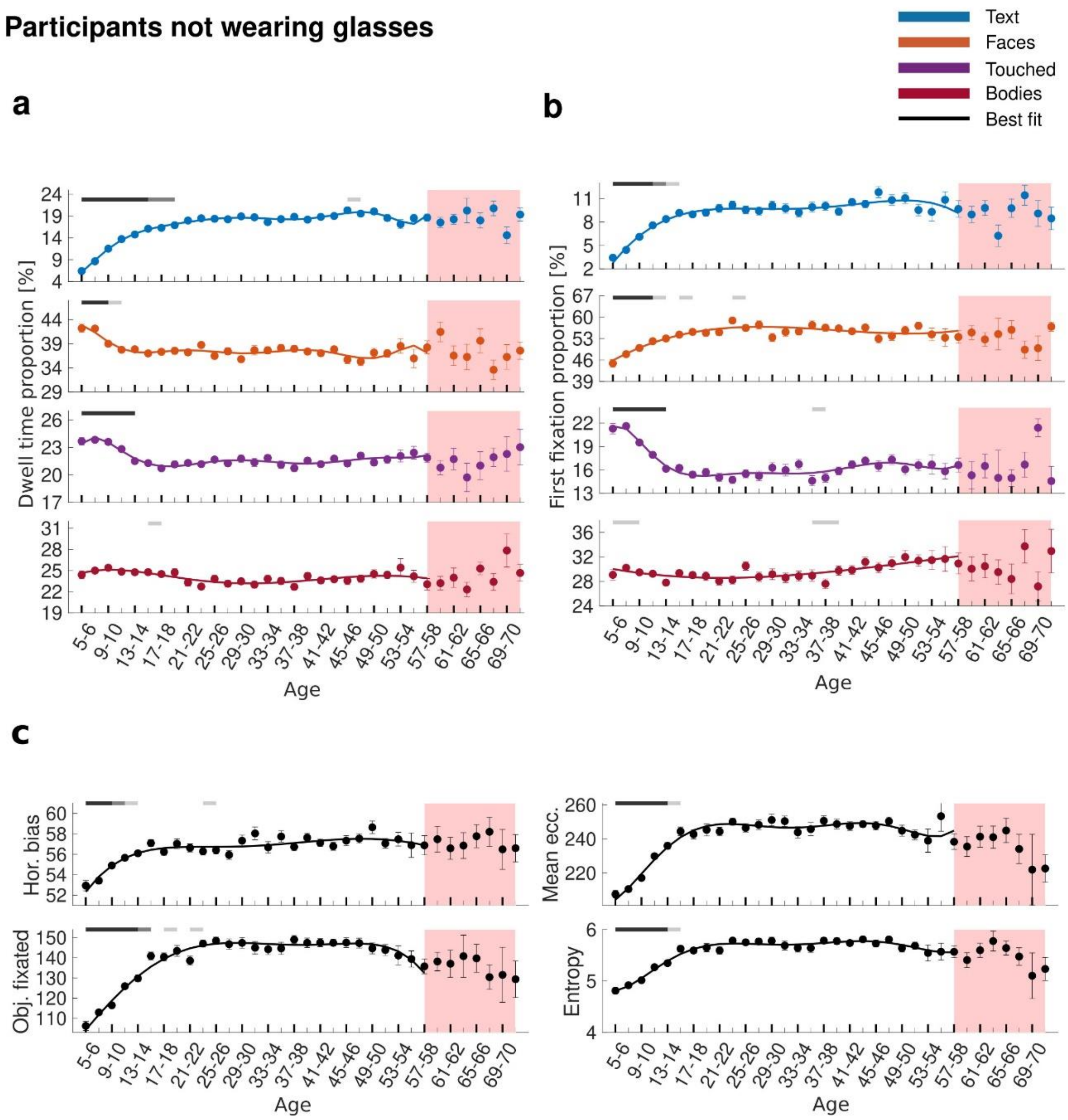

Figure S10.**Developmental trajectories excluding participants wearing glasses** Scatter points display the mean proportion of dwell time (a) and first fixations (b) directed towards objects of four semantic features across age bins ranging from ages 5-6 to 71-72 (x-axis) when excluding data from participants who indicated wearing glasses. In total, data from n = 4,849 participants were analysed. Panel c shows the average proportion of horizontal fixations, mean distance to image centroid (in pixels), average number of objects fixated, and intra-individual entropy for age bins (x-axis) when excluding participants who indicated wearing glasses. Error bars indicate +/- 1 standard error of the mean (SEM), lines best polynomial fits. The horizontal lines on top indicate the level of evidence for a given bin to be part of broad linear change via shades of grey, as shown in the inset (sliding window analysis, cf. main text and methods). Please note that each age bin spans two years. Due to space constraints, only every second tick is labelled, and these are marked in bold for easier identification. Red patches mark age bins with lower sensitivity to developmental trends (n < 30 per bin).

References

1. Linka, M. & de Haas, B. OSIEshort: A small stimulus set can reliably estimate individual differences in semantic salience. *J Vis* **20**, 13 (2020).
2. Spearman, C. The proof and measurement of association between two things. (1961).
3. Xu, J., Jiang, M., Wang, S., Kankanhalli, M. & Zhao, Q. Predicting human gaze beyond pixels. *J Vis* **14**, (2014).
4. Broda, M. D. & de Haas, B. Individual differences in looking at persons in scenes. *J Vis* **22**, 9 (2022).
5. Nyström, M. & Holmqvist, K. An adaptive algorithm for fixation, saccade, and glissade detection in eyetracking data. *Behav Res Methods* **42**, 188–204 (2010).
